# Compression-based Facies Modelling

**DOI:** 10.1007/s11004-023-10048-y

**Published:** 2023-02-23

**Authors:** Tom Manzocchi, Deirdre A. Walsh, Marcus Carneiro, Javier López-Cabrera

**Affiliations:** iCRAG and Fault Analysis Group, UCD School of Earth Sciences, University College Dublin, Dublin 4, Ireland

**Keywords:** Facies model, Connectivity, Amalgamation ratio, Compression algorithm

## Abstract

Simple object- or pixel-based facies models use facies proportions as the constraining input parameter to be honored in the output model. The resultant interconnectivity of the facies bodies is an unconstrained output property of the modelling, and if the objects being modelled are geometrically representative in three dimensions, commonly-available methods will produce well-connected facies when the model net:gross ratio exceeds about 30%. Geological processes have more degrees of freedom, and facies in high net:gross natural systems often have much lower connectivity than can be achieved by object-based or common implementations of pixel-based forward modelling. The compression method decouples facies proportion from facies connectivity in the modelling process and allows systems to be generated in which both are defined independently at input. The two-step method first generates a model with the correct connectivity but incorrect facies proportions using a conventional method, and then applies a geometrical transform to scale the model to the correct facies proportions while retaining the connectivity of the original model. The method, and underlying parameters, are described and illustrated using examples representative of low and high connectivity geological systems.

## Introduction

Facies modelling algorithms are often conceptualized on a chart which compares their ability to generate geologically realistic systems with their ability to honor observational data recorded at wells (e.g., Fig. a, after Pyrcz et al. (2015)). Hence, Sequential Indicator Simulation (SIS) is generally considered less geologically realistic than other pixel-based methods such as Truncated Gaussian (TGS), Plurigaussian, or the SNESIM Multiple-Point Statistics method, but all can include conditioning well data easily. Pattern-based MPS and Object Based Modelling (OBM) algorithms are considered to be able to produce more realistic models than the pixel-based methods, since geologically realistic shapes are defined at input either directly or through inclusion in the traning image. However, these methods are harder to condition to well data, and computationally demanding optimization methods are required to do so (Rongier et al. 2017; Wang et al. 2018). Process-based and rule-based methods are approaches in which depositional objects are placed in a stratigraphic order according to geometrical or physical rules and governed by the evolving topography of the system (Pyrcz et al. 2015; Alpak and Xue 2022). Since it is straightforward to couple facies in this approach (for example, by placing a shale drape over a sand body as part of the same depositional event) these methods can generate extremely realistic systems, but like object-based models, models generated using these methods require complicated optimization schemes for conditioning to well data (Jo et al. 2020).

**Fig. 1:**
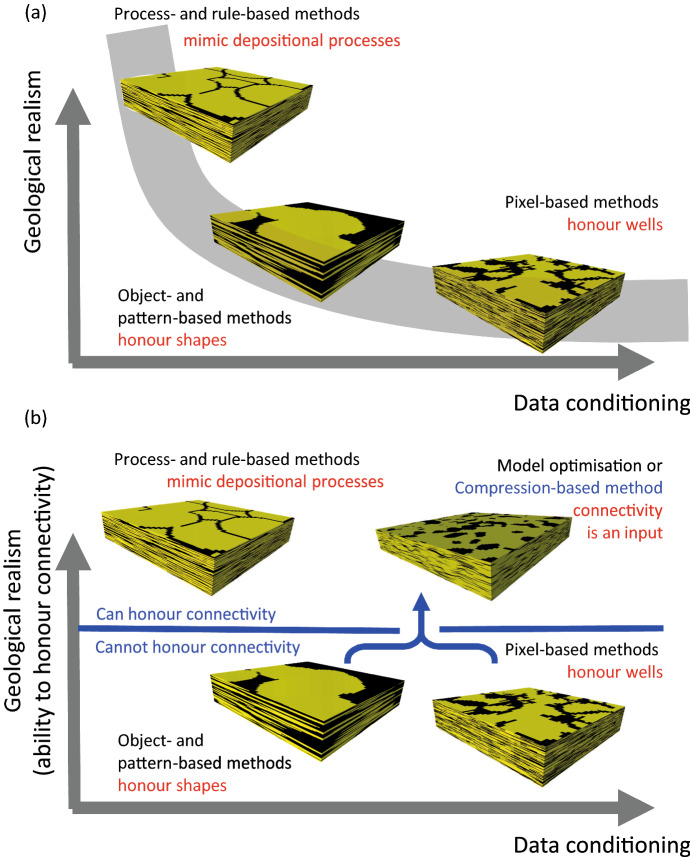
Conceptual schemes for rationalizing the ability of different facies modelling approaches to honor geological details. See text for discussion

In this paper, geological realism is expressed using a criterion related to facies connectivity. Section 2 discusses facies connectivity in natural geological systems and within object-based, pixel-based and rule-based facies models. With respect to honouring connectivity, the discussion concludes that it is inappropriate to consider within commonly available software implementations a gradual increase in geological realism from pixel-based to process-based methods, as indicated by the grey line in Fig. 1a. Instead, there are two classes of commonly available forward modelling algorithm: those in which a geologically realistic user-defined facies connectivity can be reproduced in the output model, and those in which it cannot (Fig. 1b). It appears that rule-based methods have an extra degree of freedom relative to object-based or commonly available pixel-based methods which allows them a diverse range of connectivity behavior that cannot be created with conventional object-based or pixel-based methods.

Connectivity in geological systems can be conceptualized and measured in many different ways (Renard and Allard 2013), and this study is concerned with the connectivity of geological elements within representative, stationary models containing many hundreds of elements. We are not interested in the connectivity between any two specific elements in the model, but rather on the connectivity of all the elements in a statistical sense. Model optimization and inversion methods similar to those used to condition object-based models to hard well data are able to perturb models towards specific connectivity observations contained in training images or observed between pairs or groups of conditioning wells (Laloy et al. 2017; Razak and Jafarpour 2020). This paper, however, addresses connectivity as part of the forward modelling workflow using Compression Based Modelling (CBM) rather than as an inverse optimization step. In the CBM method, a geometrical transformation is applied to a conventional facies model allowing the connectivity of the facies to be defined as a user-defined input variable (Manzocchi et al. 2007). The assumption of stationarity is central to the CBM approach, since the transformation is defined on the basis that individual vertical sections through the model have the model average properties. However, natural depositional systems may seldom be stationary and gradual depositional trends or abrupt facies transitions are likely to be present over the length-scales required for defining representative properties. For example, deep-water channel systems often contain sand-rich channel axes bounded by more shale-prone margins, and Soni et al. (2020) compared different transformations for these regions when applying CBM to this kind of system. A form of non-stationarity addressed in the current paper is the presence of hierarchical depositional elements (Prélat et al. 2009; Cullis et al. 2018), with different transformations applied in the different hierarchical objects.

The CBM method was originally devised for use with OBM, and with facies connectivity measured using the Amalgamation Ratio (AR). The objectives of the current paper are to illustrate the CBM method using: (1) different underlying geostatistical methods (TGS as well as OBM); (2) different measures of connectivity (a percolation threshold-based criterion as well as AR); (3) different conceptual geological models (low connectivity sedimentary deposits, high connectivity veins) and (4) conditioning to well data. Section 2 discusses connectivity in models and natural systems and, following Walsh and Manzocchi (2021a), describes the relationship between AR and the proximity of the system to its percolation threshold (parameter *P*, after Sahimi 1995; Renard and De Marsily 1997). Section 3 uses CBM to model a hierarchical OBM of a poorly connected deep water lobe system constrained by AR, while Sect. 4 applies it to create a TGS model of a well-connected vein system defined by a high value of *P*. In Sect. 5 the CBM method, including the well-conditioning workflow devised by Walsh and Manzocchi (2021b), is applied within a commercial geomodelling software package.

## Connectivity in Facies Models and Natural Depositional Systems

### Global Connectivity in Object-Based and Pixel-Based Models

The global connectivity of an OBM is best expressed with reference to the percolation threshold of the system. For a binary system consisting of a permeable facies within an impermeable background, the percolation threshold is usually expressed as a critical net:gross ratio (NTG$_C$) above which a continuous connected cluster (in principal of infinite extent) exists within the permeable facies, and below which it does not. NTG$_C$ has been characterized for a number of object types, and only a very brief summary is provided here. NTG$_C$ = 0.28 for three-dimensional systems of aligned cuboids (King 1990), and the threshold is similar for spheres and aligned ellipsoids (Baker et al. 2002). Introducing variably sized objects has little effect on their percolation threshold (Consiglio et al. 2003), and the anisotropy of the objects has no effect on it so long as the objects remain aligned with each other. Hence NTG$_C$ $\approx$ 0.28 for flat-lying disks, ellipsoids and semi-ellipsoids (common shapes in OBM of lobate or sheetlike sedimentary deposits) but if the ellipsoids are misaligned, increasing either their orientation distribution or their aspect ratio decreases the value of NTG$_C$ systematically (Garboczi et al. 1995; Manzocchi et al. 2007).

These studies have all examined finite sized objects within stationary three-dimensional systems many times larger than the objects in all directions, and hence the systems are geometrically representative in three dimensions. Stationarity and geometrical representivity are necessary conditions for defining a percolation threshold but are not always applicable to natural geological systems. For example, Larue and Hovadik (2006) examined channel systems which do not satisfy this condition since the channel lengths exceed the size of the system containing them. If the channels are straight and parallel to each other, the system percolates at the two-dimensional threshold (NTG$_C$ = 0.66) since the system is geometrically representative only in two directions. However, inclusion in the models of variable orientations or channel sinuosity results in a system representative in three dimensions with a threshold at NTG$_C$ $\approx$ 0.2 (Larue and Hovadik 2006).

Percolation thresholds of models generated using pixel-based methods have not received as much attention as of object-based methods, but a recent systematic study (Walsh and Manzocchi 2021a) established the three-dimensional thresholds of representative isotropic models built using an industrial implementation of the SIS, TGS and SNESIM MPS methods with different variograms (or training images). The study concluded that these pixel-based models have NTG$_C$ $\le$ 0.28, with the simplest algorithms having NTG$_C$ $\approx$ 0.13. Different versions of TGS can be defined with higher or lower thresholds (Zinn and Harvey 2003; Walsh and Manzocchi 2021a), but in all cases have NTG$_C$ $\le$ 0.28. The SNESIM model results are particularly interesting, since connectivity of MPS models had not previously been examined in detail. Srivastava (2018) showed that if a complete geostatistical description of a target image is provided, a pixel-based approach, in theory, can precisely recreate the image. Therefore, pixel-based MPS algorithms arguably should be able to honor all aspects of the training image. However, it is accepted that MPS models built using practical implementations of the SNESIM method do not necessarily reproduce the connectivity of their training images (Strebelle 2012, 2021; Tahmasebi 2018). Walsh and Manzocchi (2021a, 2021b) showed that geometrically representative three-dimensional isotropic models built using a widely available SNESIM implementation (Schlumberger 2017) are well connected if NTG > 0.28, even if the training image has very low connectivity.

### Local and Global Connectivity in Natural Systems and Facies Models

Establishing whether or not a particular system is above or below the percolation threshold relies on characterizing the size or extent of the largest cluster of connected objects in the model. The proportion of objects contained in this cluster changes from virtually none, to virtually all, between NTG values below and above the threshold, and the extent of the largest cluster changes from finite to infinite. Neither of these global characteristics can be estimated for natural geological systems based on outcrop or well data, since the full three-dimensional distribution of the objects and their interconnections is unknown. Therefore, a more local measure of connectivity is required, and the amalgamation ratio (AR) is often used. AR is defined as the proportion of object bases that overlie another object (as opposed to the inter-object facies) and can be measured in object-based and rule-based facies models as well as in natural depositional systems (Stephen et al. 2001; Manzocchi et al. 2007; López-Cabrera and Manzocchi 2019). However, AR cannot be measured in pixel-based facies models since it is an object-centric measure and pixel-based models do not contain objects.

**Fig. 2:**
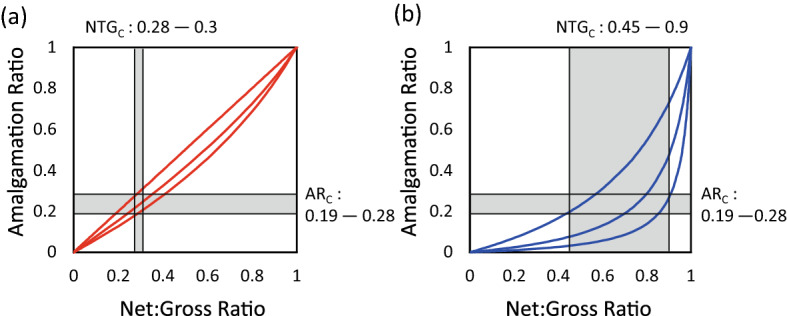
The relationship between amalgamation ratio and net:gross ratio for **a** object-based models and **b** natural deep marine deposits and rule-based models

It is well established than in an OBM containing objects of equal thickness, AR = NTG (Manzocchi et al. 2007; Walsh and Manzocchi 2021a). Therefore, an OBM of equal-sized ellipsoids, which has NTG$_C$ = 0.28, also has critical amalgamation ratio (AR$_C$) = 0.28. NTG$_C$ is relatively insensitive to the size distribution of the objects (Consiglio et al. 2003) but AR$_C$ is sensitive to size distribution because if the objects have variable thickness, AR < NTG since the thicker beds have the potential to entirely erode thinner ones. Relationships between AR and NTG for mixtures of equal proportions of beds of two different thicknesses calculated analytically are shown for an idealized OBM (Fig. a). This calculation was made by establishing the probability distribution of the distance of bed bases above and below the bases of other beds and determining from this the resultant likelihood that they are amalgamated, unamalgamated or eroded out of the system Manzocchi and Walsh (2022). A summation of probabilities provides the final AR value. The 1:1 relationship (Fig. 2a) is obtained in models in which all objects are equal thickness, and the two curves represent cases with equal proportions of two types of object with one type three or ten times thicker than the other. Results indicate that that OBM containing equal proportions of objects of two different thickness, which have NTG$_C$ in the range 0.28 to 0.30 (Consiglio et al. 2003), must have AR$_C$ in the range 0.19–0.28 (Fig. 2a).

Compilations of AR as a function of NTG for natural depositional systems consistently have AR < NTG, with different systems or hierarchical levels defining particular trends between NTG and AR similar to the lines shown in Fig. 2b (Manzocchi et al. 2007; Romans et al. 2009; Zhang et al. 2015; Soni et al. 2020). More erosive and channelized systems tend to have higher AR at a particular NTG, and lower energy sheet-like systems have lower AR. In all systems examined, AR is significantly lower than the relationships observed in OBM (Fig. 1a), which therefore do not contain realistic values of AR at particular values of NTG.

A study by López-Cabrera and Manzocchi (2019) examined a series of rule-based models of deep-water lobe systems. All models had approximately the same overall NTG (0.8) but because the models were generated with different rules governing the deposition and erosion of sand-prone and shale-prone facies, they have very different connectivity characteristics. As expected (Fig. 1a), these RBMs appear from a qualitative perspective to be much more geologically realistic than equivalent object- or pixel-based models. However, they are also more realistic from a quantitative perspective, since wells drilled in individual models follow very similar trends between local AR and NTG as the natural systems (Fig. 2b), with models generated using different rules following different trends. Hence, both natural systems and rule-based models have variable relationships between AR and NTG, with AR NTG in many cases. López-Cabrera and Manzocchi (2019) examined the global connectivity as well as the AR of the models, and found a clear percolation threshold in their models at AR$_C$ $\approx$ 0.25. Hence, the rule-based models have the same percolation threshold as the OBM in terms of AR, but a much more varied behavior in terms of NTG.

### Discussion

A number of key observations can be made from the studies discussed above. First, facies in OBM containing flat-lying ellipsoidal objects become connected in three dimensions at NTG$_C$ in the range 0.28–0.3. This corresponds to AR$_C$ in the range 0.19–0.28 (Fig. 2a). Facies in common implementations of pixel-based models become connected at similar, or lower values of NTG$_C$. Second, natural depositional systems and rule-based models have diverse relationships between AR and NTG (Fig. 2b). However, in OBM the two properties are approximately equal, and it is impossible to generate OBM with AR NTG (Fig. 2a). Third, RBMs become connected at a similar AR$_C$ value to OBMs. However, this occurs over a wider range of higher NTG values (Fig. 2b). It is likely that natural depositional systems, as well as rule-based models, become connected at NTG$_C$ in the range 0.45–0.9. These points lead to the conclusion that simple OBM are constrained by an artificial link between AR and NTG which is not present in RBM or natural systems. Since they have similar (or lower) NTG$_C$ values, pixel-based models built using two-point statistics (SIS, TGS) also suffer a similar constraint, as do the MPS models built using the industrial implementation of SNESIM examined by Walsh and Manzocchi (2021a). Put simply, these methods or implementations do not contain sufficient degrees of freedom to allow models with natural diversity of facies connectivity at high NTG values to be built.

Objects in rule-based models are stacked in depositional order according to geological rules, and the resultant models are much more representative of natural systems than the object- or pixel-based models discussed above. The ability to vary the depositional and erosional rules provides the added degree of freedom relative to OBM to model realistic connectivity. However, RBMs are problematic because they are difficult to condition to hard data (Fig. 1). The plurigaussian method (Armstrong et al. 2011) is a pixel-based approach which uses truncations of two or more correlated Gaussian fields to define the facies distribution. Use of several random fields within the same model permits a wide range of connectivity behaviour to be achieved. The method, however, has yet to receive widespread implementation in commercial software. Therefore, the current work focuses on compression-based modelling which offers a means for improving connectivity within simpler modelling workflows that can more easily honor well data, since compression-based modelling can be used in connection with simpler pixel-based methods (Walsh and Manzocchi 2021b).

### The Compression Method for Object- and Pixel-Based Models

The compression algorithm was devised initially to improve the representation of connectivity in OBM, by providing the extra degree of freedom required to allow NTG and AR to be independent inputs (Manzocchi et al. 2007). The algorithm has recently been described in detail (Walsh and Manzocchi 2021a), and consists of the two key steps. In Step 1, a model is built with the target connectivity. In the case of an OBM containing constant sized beds, this model will have an initial NTG equal to the target AR. In Step 2, the thickness of all the grid cells is scaled according to facies-specific compression factors. This does not alter the topological properties of the model, and hence both the local and global connectivity are unaffected. However, the object thicknesses and overall model NTG are modified.

**Fig. 3:**
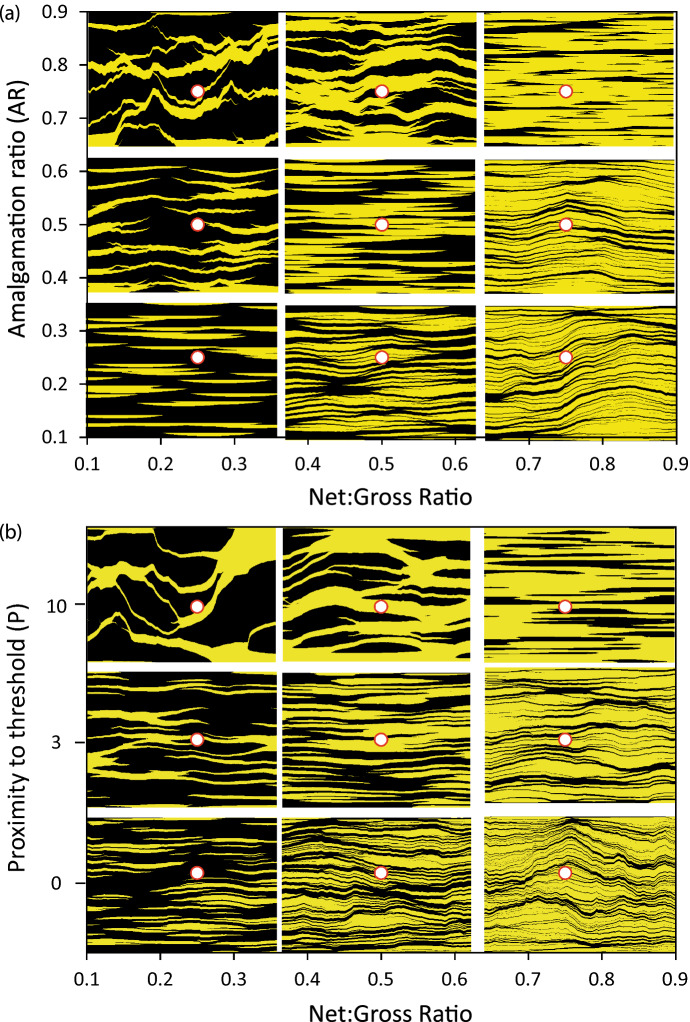
Two-dimensional CBMs at specific NTG and connectivity defined as a function of AR or *P*, generated using **a** OBM and **b** TGS. The red dots show the values used to generate the cross-sections underlying them. Sand is yellow, shale is black

The approach can be used in conjunction with object-based modelling to generate systems with independent, user-defined values of AR and NTG (Fig.a). The three systems with AR = NTG (Fig. a) are representative of conventional OBMs. The three systems that plot below these models are less connected than a normal OBM (i.e. they have AR < NTG) and are representative of low energy depositional systems as discussed in the previous sections. The three systems that plot in the upper left region (Fig. 3a) are better connected than a conventional OBM. These may also be of geological relevance since they are reminiscent of fluid-driven geological systems such as sand injectites or diagenetic vein systems (Hurst and Cartwright 2007; Meng et al. 2018).

In the CBM approach applied to an OBM (Fig. 3a), all the models were initially generated from a conventional OBM with an initial net:gross ratio (NTG$_I$) equal to AR. For the models in which AR > NTG, the cells containing shale are expanded and the cells containing sand are compressed to produce the final model, and the opposite occurs for the models with AR < NTG. The ratio between the final thickness of the shale and sand cells is the compression factor ($c_F$), and is given by Manzocchi et al. (2007); Walsh and Manzocchi (2021a)

1 $$\begin{aligned} c_{F} = \frac{{\left( {1 - \frac{1}{{{\text {NTG}}}}} \right) }}{{\left( {1 - \frac{1}{{{\text {NTG}}_{I} }}} \right) }}. \end{aligned}$$

A conventional OBM has $c_F$ = 1, but many depositional systems have $c_F$ in the range 0.03–0.3 (Fig. 2b). The initial thickness of the objects ($T_I$) scales with the final thickness (*T*) according to the relationship

2 $$\begin{aligned} T_{I} = \frac{{{\text {NTG}}_{{{\text {I}}}}T}}{{{\text {NTG}}}}. \end{aligned}$$

Amalgamation ratio is not a useful measure of connectivity in pixel-based models, and Walsh and Manzocchi (2021a) defined an equation for including connectivity as a function of the target proximity of the system to its percolation threshold (*P*). In this case, NTG$_I$ is given by

3 $$\begin{aligned} {\text {NTG}}_{I} = 1 - (1 - {\text {NTG}}_{C} )^{{P + 1}}, \end{aligned}$$

where NTG$_C$ (the critical net:gross ratio) is a variable that depends on the specific pixel-based method used to generate the model (Walsh and Manzocchi 2021a). *P* is a well-known property in percolation theory, and many physical properties of a system (such as strength or permeability) are related to *P* through power-laws (Sahimi 1995).

Example models generated using a TGS algorithm for which NTG$_C$ = 0.12 (Walsh and Manzocchi 2021a) are shown in Fig. 3b, with the target vertical and horizontal variogram ranges set to the same values as the object thicknesses and widths used for the OBMs shown in Fig. 3a. Quantitatively, the nine compressed TGS models are similar to the nine compressed OBM models, and it does not seem that the OBM models are more geologically realistic than the TGS ones, apart from the precise shape of the beds in some of the low NTG models. However, most large-scale system properties are governed by the connectivity and anisotropy of the system, rather than by details of the shapes of the bodies, and these examples demonstrate that connectivity and anisotropy can both be honored in the CBM method using either an object- or a pixel-based modelling approach.

## The Compression Algorithm for Hierarchical Object-Based Models

The simple illustration of the compression algorithm in Fig. 3 is based on models containing a single permeable facies in a background shale facies. In this case it is straightforward to calculate the thickness ($T_I$) and net:gross ratio (NTG$_I$) of the objects in the initial model, and the multipliers ($E_0$, $E_1$) by which the thickness of shale and sandstone cells must be altered to achieve the target NTG, AR and object thickness (*T*) in the final model (Walsh and Manzocchi 2021a). Here, we outline how the method has been extended to deal with multiple facies within a hierarchical setting. NTG is an impractical measure when more than one foreground facies is present, and instead the facies proportion ($P_F$) is used in the discussion.

The process is based on the analytical solution mentioned earlier Manzocchi and Walsh (2022), which provides an exact answer to the expected AR values, facies proportions and thicknesses of each facies in the final model as a function of the facies proportions, thicknesses and compression factors used the generate the initial model. For the example shown here (Fig. ), the analytical solution is used to solve the inverse problem numerically, so that the input properties ($T_I$, $P_{FI}$, *E*) of each facies are calculated as a function of the target properties of the model to be created (AR, *T*, $P_F$). The method is illustrated using a high resolution two-dimensional model in the following sub-section before it is validated in the subsequent one.

**Fig. 4:**
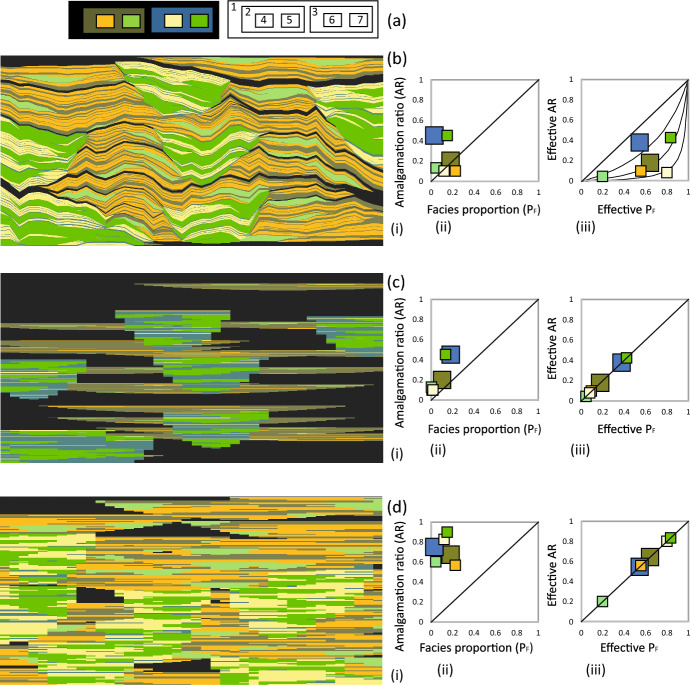
Example of a two-dimensional hierarchical compression-based model. **a** The facies hierarchy. **b** The final compression-based model. **c** The initial OBM it was generated from. **d** A comparison conventional OBM with the same hierarchy and facies proportions as **b**. In b-d, (i) shows a model cross-section, (ii) a cross-plot of AR$_T$ vs. $P_F$, and (iii) a cross-plot of AR$_E$ vs. $P_{FE}$. The curves in b (iii) are for $c_F$ = 0.03, 0.1, 0.3

### Description of the Method

Consider the hierarchical models shown in Fig. 4. These contain seven different facies consisting of two object types at each of two hierarchical levels, plus a background facies (Facies 1), as represented in the facies diagram (Fig. 4a) which shows the colors used in the model cross-sections and graphs, and the facies codes (1-7) referred to in the text. The compression-based version of the model is shown in Fig. 4b(i). At the largest scale, the model contains poorly amalgamated lobes (each of which contains Facies 2, 4 and 5) and relatively well-amalgamated channels (each of which contains Facies 3, 6 and 7). Both the lobes and the channels contain smaller-scale poorly- (Facies 4 and 6) and well-amalgamated (Facies 5 and 7) sandstone beds, while Facies 2 and 3 represent inter-bed shales.

The target facies stacking properties are defined in Fig. 4b(ii), following the approach for characterizing the stacking behavior of hierarchical systems defined in Manzocchi et al. (2020). At the largest scale, the total amalgamation ratio (AR$_T$) of object types 2 and 3 is defined by the ratio between the number of bases of each of these object types that overlie another object rather than a background shale at the hierarchical level of interest. Hence, 20% of the lobes, and 45% of the channels, are amalgamated with either a lobe or a channel (Fig. 4b(ii)). At a smaller hierarchical level, the AR$_T$ values similarly represent the probability that the objects overlie objects at the same hierarchical level, with the bright green facies (Facies 7) being the best connected at the smallest scale. These properties are loosely based on the characteristics of facies in channel lobe transition zones discussed by Fryer and Jobe (2019).

The target stacking relationships are evident in the final model realization (Fig. 4b(i)) but the propensity for the different facies to erode or aggrade is not particularly obvious from the target, facies-specific, AR$_T$ and $P_F$ values used to generate the model (Fig. 4b(ii)). This is because when more than one facies is present at a particular hierarchical level, the AR$_T$ does not follow a simple relationship with the $P_F$ (as they do for a single facies, e.g., Figs. 2b, 3) because the objects of one facies can amalgamate with objects of the other facies. This tends to result in larger facies-specific AR$_T$ values than would be present if the other facies did not exist. Additionally, the presence of more than one facies within each object type results in low facies proportions. Together, this results in many of the target AR$_T$ values of the model exceeding the target $P_F$ values (Fig. 4b(ii)). This is correct but somewhat counterintuitive.

A more intuitive representation of the stacking behavior is provided in Fig. 4b(iii), which is a cross-plot of an effective amalgamation ratio against an effective facies proportion (AR$_E$ and $P_{FE}$ respectively). These effective parameters represent the values with respect only to the facies in question and to the hierarchically equivalent shale facies. Hence, for example, the $P_{FE}$ of Facies 7 is given by

4 $$\begin{aligned} P_{{{ FE}7}} = \frac{{P_{{F7}} }}{{P_{{F7}} + P_{{F3}} }}, \end{aligned}$$

while AR$_{E7}$ is given by the number of Facies 7 object bases those overlie another Facies 7 object, as a proportion of these that overlie either Facies 7 or Facies 3. In general, AR$_E$ is marginally lower than AR$_T$ (with a larger discrepancy between the two if the facies is more poorly amalgamated) and $P_{FE}$ is significantly greater than $P_F$ (Fig. 4b(iii)).

AR$_E$ and $P_{FE}$ can be calculated as a function of AR$_T$ and $P_F$ and the other target model properties, and vice versa, which means that either set of properties could be used to define the target model properties. In this case AR$_T$ and $P_F$ have provided the target properties (Fig. 4b(ii)), but AR$_E$ and $P_{FE}$ are more intuitive graphically, since the location of a particular facies on the cross-plot can be interpreted in the same way that it could be if no other facies were present. Hence, Facies 6 is the least erosive facies in the model since it has the lowest compression factor ($c_F$ $\approx$ 0.03; Fig. 4b(iii)). This is reflected in the model realization by the common occurrence of continuous inter-beds (Facies 3) between the Facies 6 beds (Fig.4b(i)). In contrast, the large-scale channels (Object 3) are the most erosive since they have a high $c_F$ value ($\approx$ 0.5).

The way the compression algorithm works with multiple hierarchical facies, and the way the facies stacking properties scale, is perhaps easier to understand with reference to the initial OBM model generated in Step 1 of the compression modelling workflow (Fig. 4c) and to a comparison OBM generated with the same facies proportions and object thicknesses as the target model, but created using a conventional OBM workflow and hence without reference to connectivity and amalgamation (Fig. 4d). Shale Facies 1 and 3 are over-represented in the initial model (Fig. 4c(ii)) relative to the final model (Fig. 4b(ii)) since the thickness of the cells containing these facies is reduced during application of the compression algorithm. Sandstone Facies 4 and 6, by contrast, have very low proportions in the initial model and are expanded significantly in accordance with their poor connectivity. Shale Facies 2 ends up with a similar proportion in the initial and final models since it is expanded as part of the poorly amalgamated lobes but compressed because it is a shale inter-bed facies between poorly connected objects (Facies 4 and 5).

Importantly, the total and effective ARs of the initial and final model are identical (Figs. 4b, 4c). These properties govern the system topology and the compression algorithm, by design, does modify model topology. The 1:1 relationship between AR$_E$ and $P_{FE}$ for all facies in the initial model and the comparison OBM (Figs. 4c(iii) and 4d(iii)) reflects the point made earlier that a conventional OBM of constant sized objects has AR = NTG (Fig. 2a).

The facies in the comparison OBM (Fig. 4d) have identical $P_F$ and $P_{FE}$ values as the compression-based model (Fig. 4b) but much higher AR$_T$ and AR$_E$ values. Hence, although the AR$_T$ values of the facies in the hierarchical compression model are larger than their $P_F$ values (Fig. 4b(ii)), they are much lower than the equivalent values of the conventional OBM (Fig. 4d(ii)). The values are least dissimilar between the two models for the larger-scale channels (Object type 3, containing Facies 3, 6 and 7), and this is reflected by the fact that the shapes of these objects have not needed a large alteration between the initial and final models to account for their connectivity characteristics (i.e., as discussed above, they have a high $c_F$ value of $\approx$ 0.5; Fig. 4b(iii)). An artefact of CBM which is evident from comparing the initial and final models (Figs. 4c(i), b(i)) is the imposition of gradients on initially flat objects. This is an inevitable consequence of applying different compression factors across lateral facies transitions, and has been discussed by Manzocchi et al. (2020) and Walsh and Manzocchi (2021a).

### Validation of the Algorithm

The model discussed above (Fig. 4b(i)) illustrates the main aspects of the compression method when applied to multiple object types in a hierarchical OBM of a deep marine deposit, with different facies requiring different geometrical transformations to account for their connectivity characteristics (Fig. 4b(iii)). In the approach, the target properties (Fig. 4b(ii)) are used to define analytically the properties (facies proportions and object thicknesses) of an initial conventional OBM (Fig. 4c(i)) as well as the amount by which the thickness of the cells containing each facies are expanded or compressed to transform it into the final model (Fig. 4b(i)).

A validation of the approach requires that the final properties of the model reproduce the target properties used to generate it. Leuangthon et al. (2004) defined a set of minimum acceptance criteria for model validation. In this approach, aspects of a realisation (conditioning data, property distributions and mean values, variograms) are compared to their expected values. The criteria are designed for models of continuous properties rather than discrete facies for which single values of $P_F$ and AR$_T$ are obtained for each facies in an individual model realisation. Therefore, a slightly different approach to model validation is applied here. Rather than focusing on the distribution of properties obtained in a single realisation, the distribution of values of $P_F$ and AR$_T$ obtained in a series of realisations are compared to their target values. Hence, the cross-plots in Figs. a, b compare the target value to the mean value of 20 realisations, with the error bars (many of which are smaller than the symbols) reflecting the variability across the realisations (± one standard deviation).

**Fig. 5:**
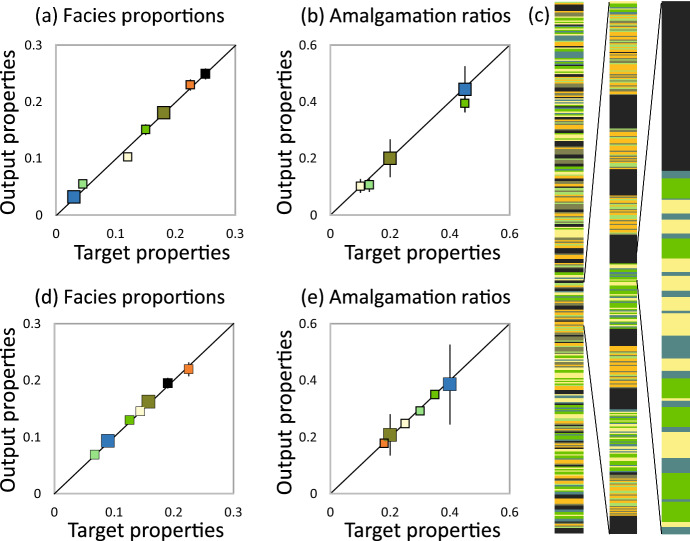
Cross-plots of target values and measured values in 20 realizations of **a** $P_F$ and **b** AR$_T$ of the two-dimensional model (e.g., Fig. 4**b**). **c** The high resolution one-dimensional model and two enlargements of it. (d, e): as a-b, but for 20 realizations of the one-dimensional model. Colors and facies hierarchy as Fig. 4

The analytical solution is based on the assumption of complete representivity, and therefore is correct if all objects are vanishingly small in relation to the hierarchically larger object they are contained in, and infinitely large in relation to the size of the modelling grid cells. Of course, these assumptions are not met in the model, and it is likely that the discrepancies (Figs. 5a, b) are due to modelling biases introduced due to non-representivity. To verify this claim, a high-resolution one-dimensional model was built (Fig. 5c) in which representivity was addressed by ensuring that the thickness of each container is at least 20 times larger than of the beds within it, and that the facies proportions used ensure that at least 20 objects are expected in each container. Additionally, the one-dimensional model was created in a continuum to eliminate grid-related biases. Results from 20 realizations (each containing about 14,000 beds) are shown in Figs. 5d, e and confirm that when representative models are built, the analytical basis of the compression methods for multiple hierarchical facies is sound.

## Practical Implementation of Compression-Based Modelling

The previous sections have provided a summary of recent work on the compression algorithm. The mathematics of the algorithm when applied to hierarchical models with several facies at each level is now established, as discussed in the previous section and described in detail by Manzocchi and Walsh (2022). That work focuses on the Amalgamation Ratio as the fundamental measure of connectivity, and hence the method is better developed for hierarchical object-based rather than pixel-based models (Manzocchi et al. 2020; Soni et al. 2020). However, definition of the relationship between AR and connectivity measures based on percolation theory (Sect. 2.4, Walsh and Manzocchi 2021a) allows pixel-based CBMs to be built as a function of user-defined connectivity. For example, Fig. shows a TGS CBM model of highly connected diagenetic gypsum veins developed during exhumation of the evaporite-rich Mercia Mudstone Formation (Meng et al. 2018), with connectivity constrained by *P*.

**Fig. 6:**
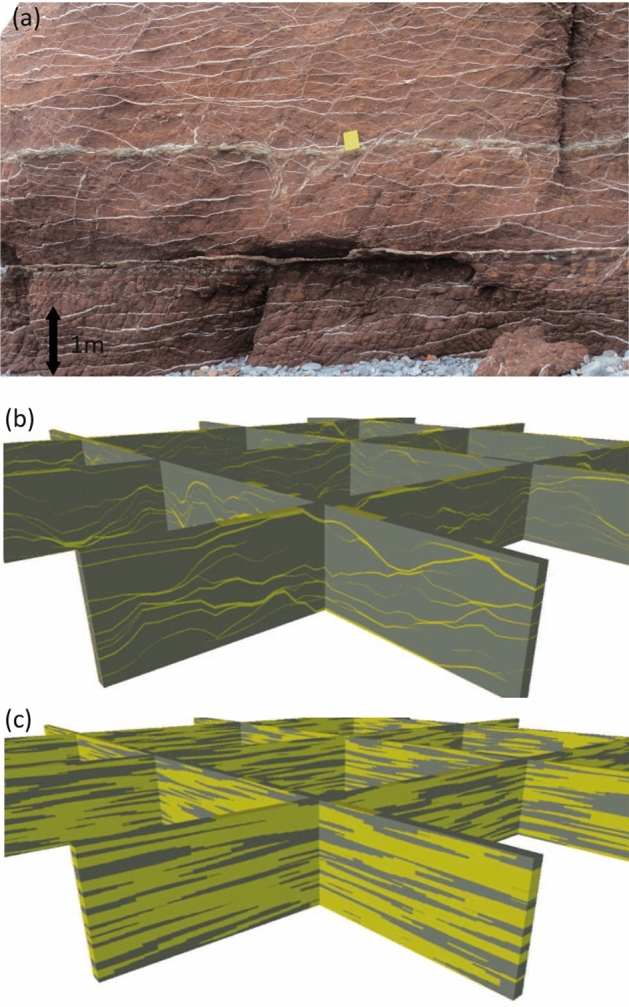
Crack-seal gypsum veins at Watchet, UK. **b** Fence diagram of an extremely well-connected CBM model built to represent one layer of the vein network (NTG = 0.09, *P* = 6). **c** The initial conventional TGS model from which the CBM model was derived (NTG$_I$ = 0.57)

In parallel to these analytical developments, work has also focused on simplifying the implementation of the hierarchical CBM approach (Walsh and Manzocchi 2019; Soni et al. 2020). A previous large-scale implementation of CBM used a stand-alone code in which nested grids were compressed individually and reassembled in a complex procedure that honored onlap and truncations at scales smaller than the grid resolution (Zhang et al. 2015; Manzocchi et al. 2020). Although flexible and geometrically accurate, that approach is extremely complicated and only suitable to object-based models. The applicability of the method has now been broadened by implementing key parts of the workflow as plugins to an industrial geo-modelling package (Schlumberger 2017, Fig. ). In this approach, the whole hierarchical facies model is built in a compressed state and then transformed in one step: an application to modelling a hierarchical submarine channel system, including a subsequent property modelling step, is described by Soni et al. (2020).

**Fig. 7:**
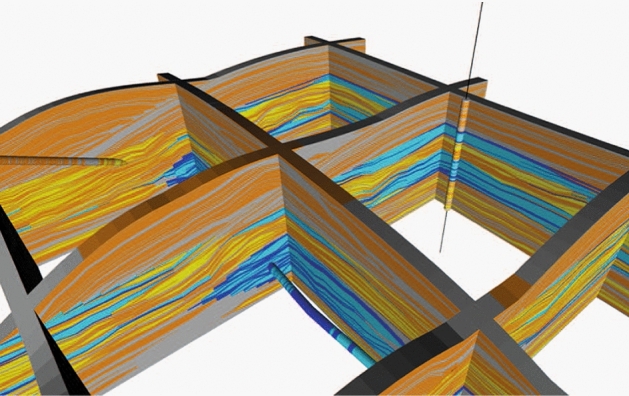
Fence diagram of a model of a synthetic sequence, generated by applying the compression workflow plugins to the pixel-based facies modelling algorithms contained in an industry-standard software package

**Fig. 8:**
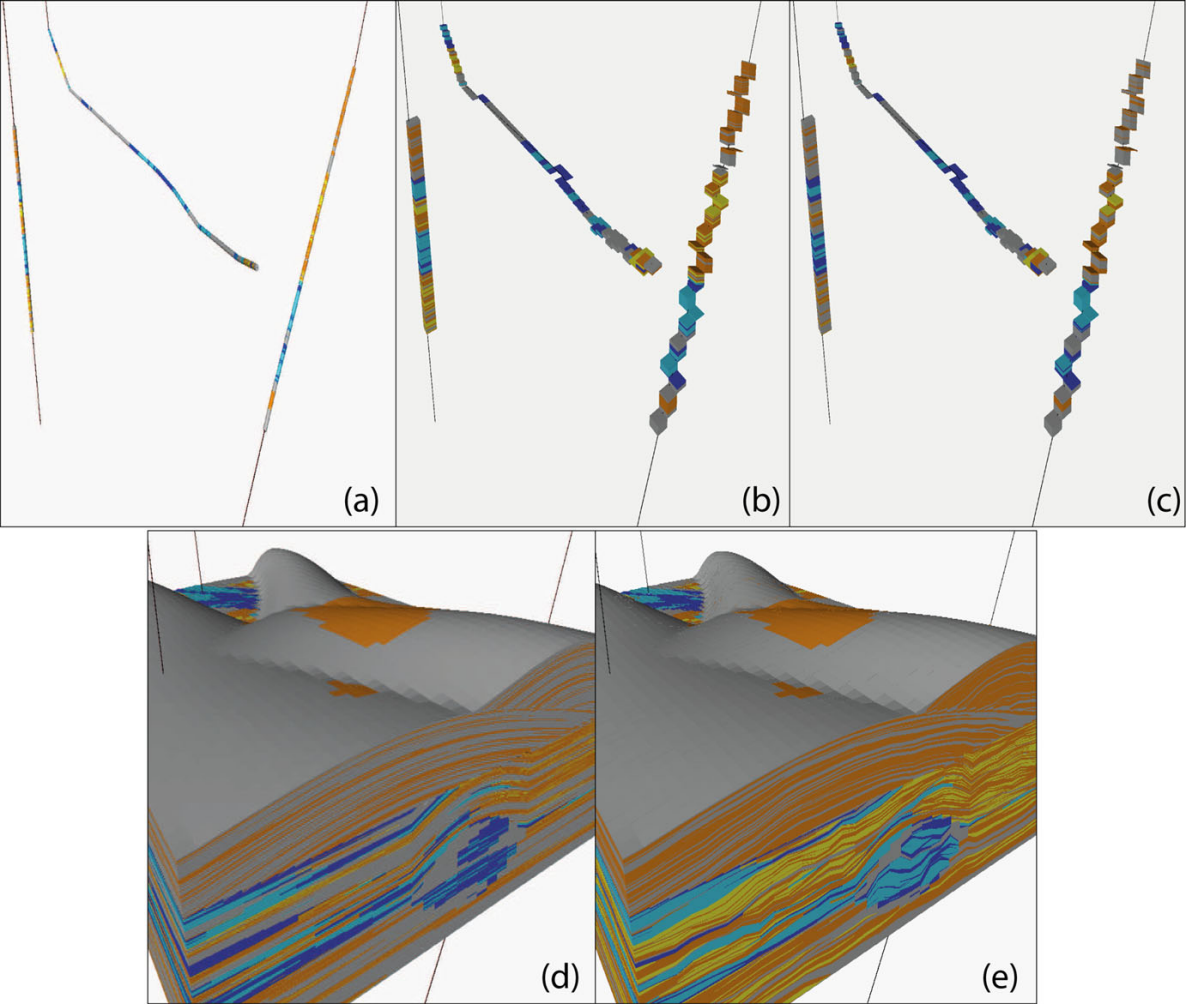
Procedure for conditioning to wells in a compression-based model. See text for details

The procedure for conditioning pixel-based CBMs to wells was described in detail by Walsh and Manzocchi (2021b). In CBM, the facies modelling is performed using a geometrical transformation of the facies description so that facies connectivity is honoured. This means that the facies thicknesses in the conditioning wells must undergo the inverse transformation prior to facies modelling. The example shown (Fig. 7) consists of multiple zones with erosive channel facies (blue) and poorly amalgamated lobe elements facies (yellow, orange) arranged hierarchically in a shale (grey) background facies and is conditioned to vertical, inclined and horizontal wells. The procedure for conditioning the model to the wells is summarised in Fig. 8. The conditioning wells (Fig. 8a) are discretised horizontally within each modelling zone at the cell stacks (Fig. 8b). A vertical transformation within each stack rescales the facies thicknesses according to the inverse of the compression factor, and the facies are then discretised vertically to the resolution of the modelling grid (Fig. 8c). A pixel-based model conditioned to the wells is then created using one of the methods available in the software (Fig. 8d). Finally, application of the compression algorithm transforms the facies model to the required facies proportions, and also restores the constraining wells to their initial condition (Fig. 8e).

## Summary and Conclusions

Compression-based modelling is a method that can be used in conjunction with object- or pixel-based facies methods to modify facies connectivity. The necessity for the approach stems from the recognition that object-based and widely available implementations of pixel-based facies modelling algorithms lack the freedom required to allow connectivity to be a user-defined input variable as opposed to an unconstrained model output. Connectivity in natural geological systems and in rule-based models is much more variable than can be represented using these methods which therefore are unsuitable for modelling a wide range of geological scenarios.

This paper has provided a snapshot state-of-the art summary of recent advances in compression-based modelling. The compression algorithm is a grid transformation that can be used to create models with independently defined facies proportions and connectivity, with the latter expressed either as a function of amalgamation ratio or proximity to percolation threshold. A workflow has been developed for defining precisely the input properties required to generate a model with target output properties for hierarchical systems with multiple objects at each level. In parallel, the approach has been implemented in an industrial geomodelling package allowing better integration with other geomodelling workflows.
